# A company doctor's role during the COVID-19 pandemic

**DOI:** 10.6061/clinics/2020/e1921

**Published:** 2020-04-30

**Authors:** Rogério Muniz de Andrade

**Affiliations:** Servico de Saude Ocupacional, Hospital das Clinicas HCFMUSP, Faculdade de Medicina, Universidade de Sao Paulo, Sao Paulo, SP, BR

The current COVID-19 pandemic poses several public health challenges. The demands placed on health services are huge, as is the need to take population protection measures. The impacts are immediately reflected on health teams. They experience increased demands and risks, such as exposure to severe acute respiratory syndrome coronavirus 2, and a compromise of their mental health.

Companies are also strongly impacted by the current situation. Some experience increased demands for their products and services, whereas others are affected by the drastic reduction in performance. Companies often employ occupational physicians, that is, specialists responsible for the health care of their employees and for ensuring a safe and healthy work environment.

Doctors working in companies must use their technical knowledge to ensure the best actions to protect the health of the workers. Concurrently, they must be aware of the work environment and the production process to suggest the best adaptations. In the current pandemic situation, the role of a doctor in a company has been strategic in ensuring the best health conditions at the workplace.

This editorial is intended at suggesting good practices to doctors working in companies. For this, it is necessary to understand the interests of employers and employees and the role of a company physician as an internal health promoter in this unique scenario.

Companies want to continue normal operations, but they need to maintain satisfactory levels of safety. Additionally, they need to manage absenteeism, as the workforce will be reduced when employees become ill or are suspected of being infected. Sometimes corporate decision makers take measures that are not in line with the best scientific evidence, with the intention of protecting workers. Companies also need to reassure their shareholders who are unsure about the fate and viability of their businesses. This is especially important when companies need to comply with government regulations by taking administrative measures that may displease their stakeholders.

All employees are concerned about their health and the health of their families. They are also concerned about safety in the workplace and have many apprehensions about their future in the company as well. This is especially true for workers who are underutilized and who are considered vulnerable to physical illness and mental health decline. In particular, workers who continue normal operations may feel a sense of injustice when they compare themselves to their co-workers who are working remotely or who are excused from their duties. Employees who continue to work may be overloaded because of the absence of other employees.

This is juxtaposed by the increased emotional overwhelm among employees who are working remotely by telecommuting. Many workers were forced to start working remotely, that is, from home, without time to organize a suitable work environment. In addition to the lack of a suitable work environment, limiting working time, family relationship time, and domestic duties becomes difficult in a remote work environment. Families need to adapt to a new routine and reorganize the tasks of children dismissed from classes in an attempt to minimize the consequences of social isolation. Telecommuters also spend a larger amount of time using electronic communication resources, which has been shown to impact mental health.

Doctors need to look for the best scientific evidence with regard to the pandemic. However, available information may be ambiguous and is constantly changing. Occupational doctors care for workers directly and also act as conflict mediators. A doctor in a company may be under pressure from several parties, including employers, employees, unions, and regulatory agencies. Doctors also need to protect their health and ensure the necessary safety measures for their work team, who are also overloaded by the increased demand from companies' outpatient clinics. A doctor's ability to adapt to frequent changes is tested during the situation.

For this reason, a company's doctor has an important role of acting as an articulator and consultant for everything related to the protection of the company's employees in the current pandemic. The doctor must ensure adequate isolation measures in the workplace and offer resources to assist patients and those suspected of being infected. The physician must clearly communicate robust health and safety policies for implementation. This is the moment that a doctor must be a health educator at all levels of the company. The doctor in a company must guarantee the participation of workers in decision-making on health action measures, which is an important element in the success of actions in occupational health. In companies that have formed a crisis committee, an occupational physician is essential to provide the necessary technical support.

The physician must establish protocols for assistance and standardized conduct for the health team, which should be changed whenever necessary on the basis of the change in knowledge on facing the crisis. The doctor must also be creative in using everyone's talents and skills, even for tasks that are not routine to a member of the healthcare team. It is time to exercise the leadership skills of company physicians and stimulate the best potential of people.

We are all learning from the current situation. We believe that these suggestions will be useful to everyone in this scenario.

